# Association between peripheral blood cytopenia and cancer mortality: A race‐specific risk factor for cancer death

**DOI:** 10.1002/cam4.5570

**Published:** 2022-12-30

**Authors:** Diego A. Adrianzen‐Herrera, Insu Koh, Radhika Gangaraju, Tomi Akinyemiju, Neil A. Zakai

**Affiliations:** ^1^ Department of Medicine Larner College of Medicine at the University of Vermont Burlington Vermont United States; ^2^ Department of Pathology and Laboratory Medicine Larner College of Medicine at the University of Vermont Burlington Vermont United States; ^3^ Department of Medicine University of Alabama at Birmingham Birmingham Alabama United States; ^4^ Department of Population Health Sciences Duke University School of Medicine Durham North Carolina United States

**Keywords:** African American, anemia, cause of death, healthcare disparity, leukopenia, neoplasms, thrombocytopenia

## Abstract

**Background:**

Cytopenia is associated with cancer through mechanisms including clonal hematopoiesis and chronic inflammation. Cytopenia is more prevalent in Black people but its relationship with racial disparities in cancer mortality is unknown.

**Methods:**

Cytopenia was defined in 19,028 Black and White participants recruited between 2003 and 2007 for the REasons for Geographic and Racial Differences in Stroke cohort, based on age‐, sex‐, and race‐adjusted ranges for blood counts. Cancer death was ascertained from Social Security Death and National Death Indexes. Multivariable Cox models estimated the risk of cancer mortality associated with cytopenia, adjusting for demographics (model1), anemia and cancer risk factors (model2), and socioeconomics (model3). Racial differences in the cytopenia‐cancer death association were tested by cross‐product interaction terms.

**Results:**

Cytopenia was identified in 383 (2%) participants, 250 (65%) White, and 113 (35%) Black people. With median follow‐up 11.3 years, 1,224 (6.4%) cancer deaths occurred. Cytopenia was associated with increased risk of cancer mortality in model1 (HR = 1.57, 95%CI 1.15–2.24), model2 (HR = 1.67, 95%CI 1.22–2.30), and model3 (HR = 1.59, 95%CI 1.17–2.17). Participants with cytopenia had twofold increased cumulative incidence of cancer death (13% vs. 6.5%, *p* < 0.01). Race by cytopenia interaction terms showed higher HR for cancer death in Black compared to White participants: 2.01 versus 1.41 (*p*_interaction_ = 0.016, model1), 2.12 versus 1.45 (*p*_interaction_ = 0.009, model2), and 1.82 versus 1.44 (*p*_interaction_ = 0.04, model3).

**Conclusion:**

In this large, observational biracial prospective study, cytopenia was a risk factor for cancer death, with stronger association in Black than White people. Though race impacted the association of cytopenia with cancer mortality, cytopenia was not a mediator of the racial disparity in cancer mortality.

## Graphical abstract

In a large biracial cohort, cytopenia was associated with increased risk of cancer mortality and constituted a race‐specific risk factor for cancer death, with stronger association in Black compared to White participants. Race was an effect modifier in the association of cytopenia and cancer mortality but not a mediator of the Black to White difference in cancer death.

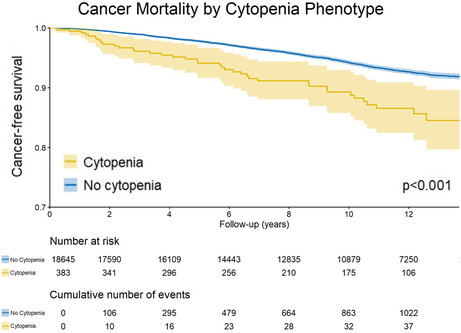

## 1 BACKGROUND

Cytopenia refers to reduced cell counts in peripheral blood and is associated with various medical conditions.^1^, ^2^ Some cytopenia cases are deemed idiopathic and a specific diagnosis cannot be established.^3^ Individuals with unexplained peripheral cytopenia remain a challenge in clinical practice^4^; they constitute a diagnostic dilemma for clinicians, who need to sort through complex boundaries and novel categories to differentiate between a wide array of etiologies, from transient abnormalities and occult medical illness to bone marrow failure syndromes and premalignant conditions.^5^, ^6^

Pathobiological mechanisms have linked blood cytopenia to increased risk of hematologic cancer.^7^ A subset of individuals with unexplained cytopenia harbor mutated hematopoietic stem cells and will develop myeloid malignancies such as myelodysplastic syndromes or acute myeloid leukemia through progression of clonal hematopoiesis.^7^, ^8^ There is also an association between cytopenia, particularly anemia, aging, and carcinogenesis.^9^ Proposed underlying mechanisms include progressive inflammatory responses causing reduction in the functional reserve of multiple organ systems, rendering individuals simultaneously vulnerable to hematopoietic stress and neoplastic transformation.^10^, ^11^, ^12^ Chronic inflammation has a negative prognostic correlation with various types of cancers and bone marrow failure syndromes^5^ and has been shown to trigger clonal evolution of neoplastic cells, including second cancers in individuals with clonal hematopoiesis.^13^ These associations result from pro‐inflammatory cellular responses across various pathways of carcinogenesis and are mostly described, but not limited to, older adults.^14^, ^15^, ^16^

Unexplained cytopenia can therefore represent an early manifestation of serious medical conditions which include or may result in cancer through shared underlying inflammatory mechanisms. Studies have linked blood cellular abnormalities, particularly declining hemoglobin concentration, with risk of developing both hematologic and solid cancer, and anemia may represent an early sign of malignant disease.^17^, ^18^ Cytopenia can precede a clinical diagnosis of cancer, particularly hematologic malignancies, by prolonged periods of time^19^ and once cancer has been established, cytopenia has been associated with increased mortality, independent of tumor type and treatment.^20^, ^21^, ^22^

Racial differences in peripheral blood cell counts are known; Black Americans frequently have lower hemoglobin and white blood cell (WBC) values, including total leukocyte and neutrophil counts, compared to White Americans.^23^, ^24^, ^25^ These differences are partly caused by inherited hemoglobin traits,^26^ ethnic variants,^27^ and socioeconomic inequalities^28^; however, the increased anemia prevalence in Black people is not fully explained by demographics, socioeconomics, or comorbidity burden.^29^ A study using the National Health and Nutrition Examination Survey (NHANES) described patterns of unexplained cytopenia in a nationally representative United States (US) sample and identified disproportionately high prevalence among Non‐Hispanic Black people.^30^ This seemingly intrinsic higher prevalence of cytopenia among Black people may constitute a marker of health disparities given the substantial evidence linking cytopenia, particularly anemia, with poorer health outcomes, including higher mortality.^31^, ^32^

Using the REasons for Geographic and Racial Differences in Stroke (REGARDS) cohort, we previously defined a cytopenia phenotype associated with increased cardiovascular and all‐cause mortality.^33^ Based on shared mechanisms linking cytopenia to cancer death and the notion of cytopenia as a marker of racial disparity, this study aimed to determine if cytopenia is associated with cancer mortality and whether it is a factor contributing to cancer death disparities in Black Americans.

## 2 METHODS

### 2.1 Population

REGARDS is an ongoing national longitudinal cohort study in the U.S. designed to evaluate factors contributing to racial and geographic differences in stroke mortality and cognitive decline. Details of REGARDS design have been previously published.^34^ Briefly, between 2003 and 2007, a total of 30,239 community‐dwelling adults aged 45 years or older were recruited using postal mailings and telephone data. Potential participants were initially contacted via mail, then by telephone for a structured interview to establish eligibility, collect basic health information, and obtain verbal informed consent. Subsequently, trained technicians (Exam Management Systems Incorporated) conducted a scripted in‐home and obtained written informed consent. The study abided by the precepts of the Declaration of Helsinki and obtained institutional review board approval from all participating institutions. Recruitment intended to oversample Black Americans and residents of the stroke belt (North and South Carolina, Georgia, Tennessee, Mississippi, Alabama, Louisiana, and Arkansas). Self‐reported races other than Black or White were excluded.

### 2.2 Study design

Upon enrollment, information about demographics, health behaviors, chronic medical conditions, and medications was collected via phone interview, which was followed by an in‐home study visit for vital signs and anthropometric measures, phlebotomy, and urine collection. Active treatment for cancer, medical conditions preventing long‐term participation, cognitive impairment, residence in nursing home, and inability to communicate in English were exclusion criteria. Participants were followed prospectively to identify medical events, hospitalizations, or death, and routine linkages with national databases were used to ascertain mortality outcomes.

### 2.3 Study population

Following the first 8,400 recruitments, complete blood count (CBC) was added to the baseline assessment. Blood samples obtained during in‐home visits were refrigerated and shipped the same day to the study central laboratory at the University of Vermont.^35^ CBC was performed from intact ethylene‐diaminetetraacetic acid tubes using automated cell counting on a Coulter LH755 Hematology Workcell (Beckman Coulter Incorporated, Fullerton, CA).^29^ To assess the association of peripheral cytopenia and cancer mortality, we analyzed data from 19,028 REGARDS participants for whom CBC at enrollment was available and included results for all hematologic values of interest: Hemoglobin, WBC, Platelet count, and mean corpuscular volume (MCV). Participants with no follow‐up were excluded or censored at the time they were lost to follow‐up (Figure S1).

### 2.4 Exposure variables

Using values specified in Table S1, cytopenia phenotype was defined as presence of two or more of: (a) hemoglobin below age‐, sex‐, and race‐specific lowest fifth percentile; (b) WBC below race‐specific lowest fifth percentile; (c) platelet count below the lowest fifth percentile, and (d) macrocytosis, defined as MCV higher than 98 fL. The decision to use age‐, sex‐, and race‐specific percentiles and race‐specific percentiles for hemoglobin and WBC thresholds, respectively, was based on the established influence of demographic characteristics on these values, while no known age, sex, or race differences are recognized for platelet count or MCV.^26^, ^36^, ^37^, ^38^, ^39^ Macrocytosis was included with the goal of defining a cytopenia phenotype with higher chance of predicting early bone marrow failure, as can be seen in clonal hematopoiesis. In participants with chronic kidney disease (CKD), defined as estimated glomerular filtration rate (eGFR) less than 60 ml/min/1.73m^2^, presence of macrocytosis was required in addition to hemoglobin below the above mentioned threshold for the definition of anemia, to account for anemia of kidney disease.^40^

### 2.5 Outcomes measures

The primary outcome was REGARDS‐adjudicated cancer‐specific mortality, from any malignant disease. Secondary outcomes included death from hematologic malignancy or solid cancer. Cancer mortality ascertainment in REGARDS has been previously reported and validated.^41^ Trained personnel identified any death during semi‐annual telephone follow‐ups. A committee of expert clinicians and investigators, who underwent specific training to identify causes of death, reviewed death certificates, and medical records, interviewed participants' proxies, and examined administrative databases to adjudicate the primary cause of death, following a previously published process.^42^ Further, cancer as primary cause of death was verified through linkages with the Social Security Death Index (SSDI) and the National Death Index (NDI) following published national guidelines. Only participants in which cancer was the primary cause of death were considered to have the outcome of interest. The validity of SSDI and NDI as an accurate method to identify cancer mortality is established.^43^, ^44^, ^45^ Participants were followed from enrollment through the date of death, loss to follow‐up, or last follow‐up as of December 31, 2018.

### 2.6 Statistical analysis

Standard descriptive statistics were used to describe participants' characteristics, using medians, ranges, frequencies, and percentages. The associations between risk factors and hematologic parameters, cytopenia, and cancer mortality was evaluated using χ^2^ tests for categorical variables and 2‐tailed *t‐*tests for continuous variables. Cox proportional hazards models were used to calculate hazard ratios (HRs) and 95% confidence intervals (CI) for cancer mortality associated with cytopenia, adjusting for confounders in three models. Model one (demographics model) adjusted for demographic factors including age, race, sex, and geographic region. Model two (anemia/cancer model) added conventional risk factors for anemia and/or cancer to model one, including history of cancer prior to enrollment, smoking status, body mass index (BMI), diabetes, alcohol consumption, C‐reactive protein (CRP) as a marker of inflammation, and eGFR as a marker of CKD. Model three (socioeconomic model) added socioeconomic risk factors to model one, including self‐reported income, insurance status, and education level. Differences in the association of cytopenia phenotype and cancer mortality by race were tested using cross‐product interaction terms. To determine the effect of cytopenia as a mediator in the race‐to‐cancer‐death interaction, mediation analysis was conducted using the inverse odds ratio weighting (IORW) method^46^ and general multiple mediation analysis,^47^ using a *p*‐value of <0.1 for interaction.

## 3 RESULTS

### 3.1 Cancer mortality

The analysis population included 19,028 participants with median follow‐up time of 11.3 years (interquartile range [IQR]: 6.5–13). Overall, 62% of the cohort were female and 60% White individuals. The mean age at enrollment was 64 years (standard deviation [SD] = 9.7). There were 4,844 deaths during follow‐up and 25.3% of them (1,224) were cancer deaths, corresponding to 6.4% participants. Of 1,224 cancer deaths, 154 (12.6%) were attributable to hematologic malignancies and 1,070 (87.4%) to solid tumors. The demographic and clinical characteristics of study participants according to their death outcome are presented in Table 1. Obesity, diabetes, current smoking or alcohol use, and previous history of cancer were more common in participants who died of cancer. Outside younger age, no other notable differences in demographic factors were identified between participants with cancer death and those who died from other causes.

**TABLE 1:** Demographic and clinical characteristics of study participants according to their death outcome

	Full cohort	Non‐cancer death	Cancer death	*p*‐Value*
	(*N* = 19,028)	(*N* = 3620)	(*N* = 1224)	
Sex, *n* (%)				
Female	11,793 (62%)	1921 (53%)	633 (52%)	0.347
Male	7235 (38%)	1699 (47%)	591 (48%)	
Age (years)				
Mean (SD)	64.0 (9.70)	71.2 (9.52)	68.7 (9.05)	<0.001
Race, *n* (%)				
White	11,445 (60%)	2119 (59%)	740 (60%)	0.344
Black	7583 (40%)	1501 (41%)	484 (40%)	
Region, *n* (%)				
Rest of the United States	7713 (41%)	1479 (41%)	519 (42%)	0.261
Stroke belt	6700 (35%)	1284 (35%)	436 (36%)	
Stroke buckle	4615 (24%)	857 (24%)	269 (22%)	
Education, *n* (%)				
High school or less	7089 (37%)	1721 (48%)	546 (45%)	0.122
Some college or more	11,924 (63%)	1888 (52%)	677 (55%)	
Missing	15 (0.1%)	11 (0.3%)	1 (0.1%)	
Annual Income, *n* (%)				
Less than $35,000	7590 (40%)	1956 (54%)	619 (51%)	0.004
$35,000 and more	8993 (47%)	1125 (31%)	438 (36%)	
Refused	2445 (13%)	539 (15%)	167 (14%)	
Rurality, *n* (%)				
Urban (≥75% urban)	13,115 (69%)	2633 (73%)	861 (70%)	0.146
Mixed (25%–75% urban)	1975 (10%)	324 (9%)	141 (12%)	
Rural (≤25% urban)	1996 (10%)	319 (9%)	119 (10%)	
Missing	1942 (10.2%)	344 (9.5%)	103 (8.4%)	
Obesity (by BMI)				
Not obese	11,474 (60%)	2223 (61%)	830 (68%)	<0.001
Obese	7420 (39%)	1351 (37%)	386 (32%)	
Missing	134 (0.7%)	46 (1.3%)	8 (0.7%)	
Diabetes, *n* (%)				
No	956 (78%)	2376 (66%)	956 (78%)	<0.001
Yes	256 (21%)	1223 (34%)	256 (21%)	
Missing	12 (1.0%)	21 (0.6%)	12 (1.0%)	
eGFR (mL/min/1.73m^2^)				
Median (IQR)	88.8 (74.3–99.7)	76.6 (58.7–90.8)	85.6 (69.6–96.4)	<0.001
Missing	31 (0.2%)	9 (0.2%)	1 (0.1%)	
C‐Reactive Protein (mg/L)				
Median (IQR)	0.784 (−0.0619–1.63)	0.975 (0.0677–1.85)	0.908 (0.113–1.71)	0.369
Missing	262 (1.4%)	51 (1.4%)	13 (1.1%)	
Smoking history, *n* (%)				
Never or past	16,206 (85%)	3008 (83%)	915 (75%)	<0.001
Current	2752 (14%)	603 (17%)	301 (25%)	
Missing	70 (0.4%)	9 (0.2%)	8 (0.7%)	
Alcohol use, *n* (%)				
Never or past	9188 (48%)	2110 (58%)	600 (49%)	<0.001
Current	9840 (52%)	1510 (42%)	624 (51%)	
Past history of cancer, *n* (%)				
No	17,851 (94%)	3285 (91%)	1066 (87%)	<0.001
Yes	1177 (6%)	335 (9%)	158 (13%)	
Insurance, *n* (%)				
No	1349 (7%)	178 (5%)	79 (6%)	0.064
Yes	17,658 (93%)	3435 (95%)	1145 (94%)	
Missing	21 (0.1%)	7 (0.2%)	0 (0%)	
Hemoglobin (g/dL)				
Median (IQR)	13.6 (12.7–14.6)	13.4 (12.3–14.5)	13.7 (12.7–14.6)	<0.001
WBC (×10^9^/L cells)				
Median (IQR)	5.65 (4.64–6.88)	5.97 (4.85–7.30)	5.92 (4.77–7.19)	0.613
Platelets (×10^9^/L cells)				
Median (IQR)	230 (192–274)	219 (177–264)	225 (183–268)	0.201
MCV (fL)				
Median (IQR)	90.0 (87.0–93.0)	91.0 (87.0–94.0)	91.0 (88.0–94.0)	0.863

Abbreviations: BMI, body mass index; eGFR, estimated glomerular filtration rate; MCV, mean corpuscular volume; SD, standard deviation; WBC, white blood cell count.

* *p*‐values represent the comparison between participants who died from cancer and those who died from other causes.

### 3.2 Cytopenia prevalence

Baseline cytopenia phenotype was identified in 383 participants, of whom 250 (65%) were White participants and 113 (35%) were Black participants. The overall prevalence of cytopenia in the study cohort at enrollment was 2% and was higher in White compared to Black participants (2.2% vs. 1.8%, *p* = 0.038). Table S2 shows the prevalence of individual hematologic parameters within the cytopenia phenotype, stratified by race. No differences were identified for anemia, leukopenia, and thrombocytopenia, but macrocytosis was more frequent in White participants (5.2% vs. 3%, *p* < 0.001). In addition, cytopenia was more common in males compared to females (56% vs. 44%, p < 0.001) and prevalence increased with age (median age 68 vs. 63 years in participants with and without cytopenia, *p* < 0.001). White men older than 65 years had the highest prevalence of cytopenia. The demographic and clinical characteristics of the study participants according to presence or absence of baseline cytopenia are shown in Table S3.

### 3.3 Cytopenia and cancer mortality

**FIGURE 1:**
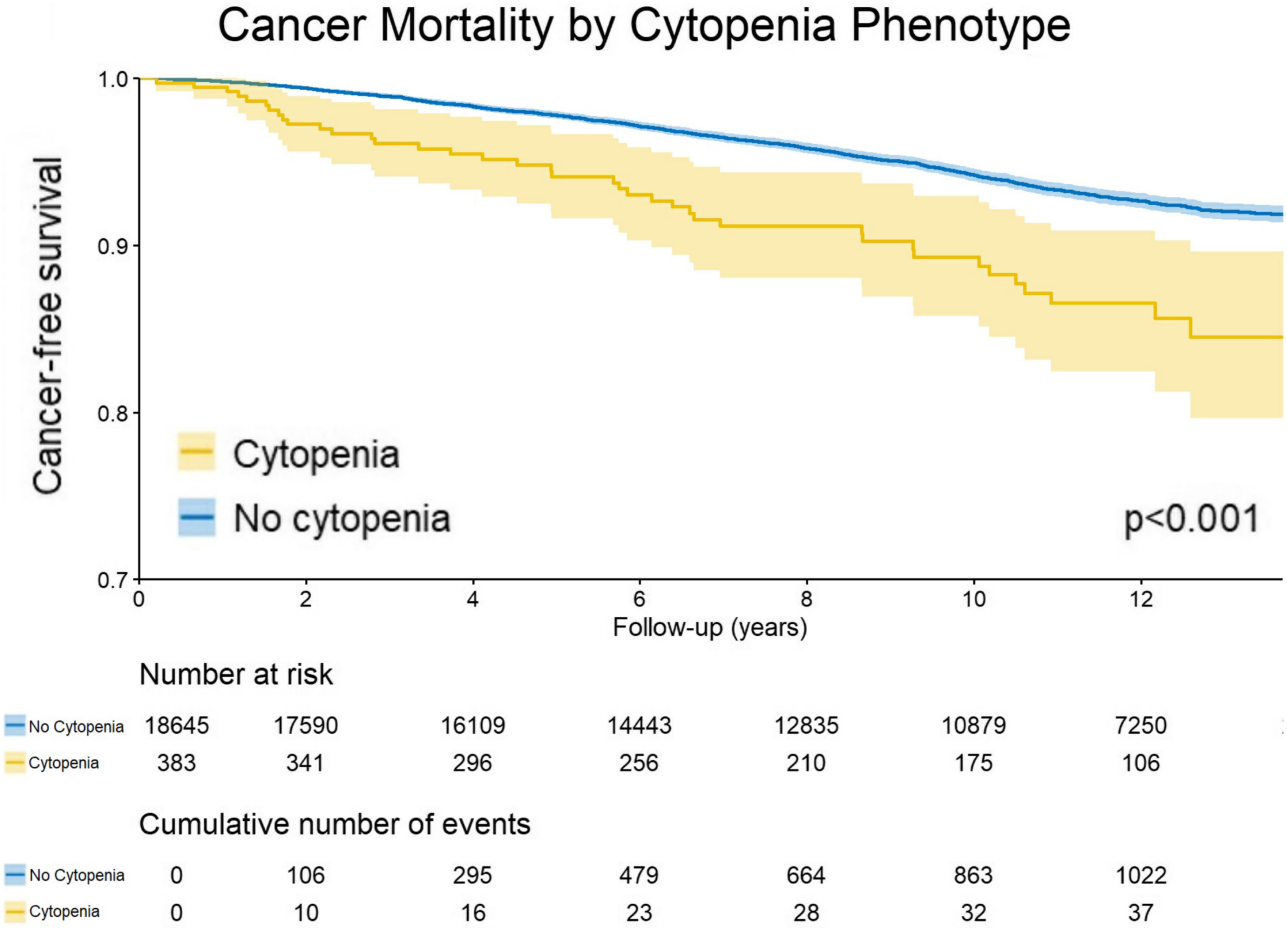
Unadjusted Kaplan–Meier survival curves with estimates for cancer‐specific survival for participants with and without cytopenia phenotype at enrollment.

Cytopenia was more frequent in participants who died from any cause (3.9% vs. 1.4%, p < 0.001) and participants with cancer death (3.4% vs. 1.8%, *p* < 0.001), compared to those alive at end of follow‐up. In regression analysis, cytopenia phenotype was associated with increased risk of cancer mortality in all multivariable models. Cytopenia was associated with 57% increased hazard of cancer death (HR = 1.57, 95%CI 1.15–2.24) in the demographics model, adjusting for demographic factors alone; 67% increased hazard of cancer death (HR = 1.67, 95%CI 1.22–2.30) in the anemia/cancer model, adjusting for demographics and risk factors for anemia and cancer; and 59% increased hazard of cancer death (HR = 1.59, 95%CI 1.17–2.17) in the socioeconomic model, adjusting for demographics and socioeconomic factors. Outside cytopenia, anemia and macrocytosis were hematologic parameters individually associated with increased hazard of cancer death across all models, while no association was identified for leukopenia or thrombocytopenia (Table 2). The 10‐year cumulative incidence of cancer death was 13% for participants with baseline cytopenia phenotype compared to 6.5% for those without cytopenia (*p* < 0.001, Figure 1). Hematologic and solid cancers were also analyzed separately. Cytopenia was associated with over fivefold increased risk of death from hematologic malignancy: HR = 5.28 in the demographic model, HR = 5.46 in the anemia/cancer model, and HR = 5.36 in the socioeconomic model. All independent hematologic parameters were significantly associated with increased risk of hematologic cancer death, with anemia showing the highest risk. Similarly, anemia and macrocytosis were associated with increased risk of death from solid cancer across all models (Table 3).

**TABLE 2:** Hazard for Cancer‐specific mortality by hematologic parameter

Hematologic parameter	Demographic model	Anemia/Cancer model	Socioeconomic model
	(HR, 95%CI)	(HR, 95%CI)	(HR, 95%CI)
Anemia	1.92 (1.56, 2.38)	1.96 (1.57, 2.44)	1.87 (1.51, 2.32)
Leukopenia	0.98 (0.76, 1.28)	1.14 (0.86, 1.50)	1.01 (0.78, 1.32)
Thrombocytopenia	1.20 (0.96, 1.49)	1.30 (1.04, 1.62)	1.16 (0.93, 1.44)
Macrocytosis	1.65 (1.33, 2.05)	1.49 (1.20, 1.85)	1.69 (1.37, 2.09)
Cytopenia	1.57 (1.15, 2.14)	1.67 (1.22, 2.30)	1.59 (1.17, 2.17)

*Note*: Model one estimates are adjusted for age, race, sex, and geographic region. Model two estimates are additionally adjusted for cancer history, smoking status, BMI, diabetes, alcohol consumption, CRP, and eGFR. Model three estimates are additionally adjusted for income, insurance status, and education level.

Abbreviations: CI, confidence interval; HR, hazard ratio.

**TABLE 3:** Hazard ratios for death from hematologic neoplasia and death from solid tumor by hematologic parameter

Hematologic parameter	Hematologic neoplasia			Solid tumor		
	Demographics model	Anemia/Cancer model	Socioeconomic model	Demographics model	Anemia/Cancer model	Socioeconomic model
	(HR, 95%CI)	(HR, 95%CI)	(HR, 95%CI)	(HR, 95%CI)	(HR, 95%CI)	(HR, 95%CI)
Anemia	3.90 (2.48, 6.14)	3.95 (2.45, 6.35)	**3.89 (2.47, 6.12)**	**1.66 (1.31, 2.12)**	**1.69 (1.31, 2.17)**	**1.61 (1.27, 2.05)**
Leukopenia	2.87 (1.79, 4.59)	3.18 (1.92, 5.26)	2.94 (1.84, 4.71)	0.74 (0.54, 1.02)	0.87 (0.63, 1.21)	0.76 (0.56, 1.05)
Thrombocytopenia	2.55 (1.62, 4.01)	2.77 (1.75, 4.37)	2.47 (1.57, 3.89)	1.01 (0.79, 1.30)	1.09 (0.85, 1.41)	0.98 (0.76, 1.26)
Macrocytosis	2.21 (1.30, 3.74)	2.16 (1.25, 3.74)	2.29 (1.35, 3.87)	1.57 (1.24, 1.99)	1.40 (1.10, 1.78)	1.60 (1.27, 2.03)
Cytopenia	5.28 (3.16, 8.82)	5.46 (3.21, 9.30)	5.36 (3.21, 8.96)	1.06 (0.71, 1.58)	1.14 (0.76, 1.72)	1.08 (0.72, 1.61)

*Note*: Model one estimates are adjusted for age, race, sex, and geographic region. Model two estimates are additionally adjusted for cancer history, smoking status, BMI, diabetes, alcohol consumption, CRP, and eGFR. Model three estimates are additionally adjusted for income, insurance status, and education level.

Abbreviations: CI, confidence interval; HR, hazard ratio.

### 3.4 Racial differences in cytopenia and cancer mortality

The race by cytopenia interaction term was statistically significant in all models evaluating cytopenia and risk of cancer death, indicating a significantly higher risk for cancer mortality associated with cytopenia in Black compared to White participants: HR of 1.82 versus 1.45 (*p*_interaction_ = 0.022) in the model adjusting for demographics, HR of 1.96 versus 1.54 (*p*_interaction_ = 0.009) in the model adjusting for demographics, anemia and cancer risk factors, and HR of 1.81 versus 1.49 (*p*_interaction_ = 0.019) in the model adjusting for demographics and socioeconomic factors. Additionally, anemia, thrombocytopenia, and macrocytosis were individual hematologic parameters associated with higher risk for cancer death in Black compared to White participants, with significant race by cytopenia interaction terms across all three multivariable models. This interaction was not identified for leukopenia (Table 4). Analysis of the race by cytopenia interaction with death from hematologic cancer or solid tumor as distinct outcomes showed that the risk of death from hematologic cancer associated with cytopenia was higher in Black compared to White participants across all models. The HRs for death from solid tumor were also higher for Black compared to White participants, though interaction terms were not significant. Additionally, significant race interaction terms were identified across models for all individual hematologic parameters in analysis of death from hematologic cancer while race interaction was only observed for anemia and macrocytosis when outcome was death from solid tumor (Table 5). Mediation analyses conducted using the IORW method and general multiple mediation analysis demonstrated that cytopenia was not a significant mediator in the pathway between the race and cancer mortality association (Table S4).

**TABLE 4:** Hazard for cancer‐specific mortality by hematologic parameter stratified by race

Hematologic parameter	Demographic model			Anemia/Cancer model			Socioeconomic model		
	(HR, 95%CI)			(HR, 95%CI)			(HR, 95%CI)		
	Blacks	Whites	*p* ^int^ ^a^	Blacks	Whites	*p* ^int^ ^a^	Blacks	Whites	*p* ^int^ ^a^
Anemia	2.12 (1.53, 2.95)	1.81 (1.37, 2.39)	<0.001	2.22 (1.58, 2.13)	1.79 (1.34, 2.40)	<0.001	2.08 (1.49, 2.88)	1.75 (1.33, 2.32)	<0.001
Leukopenia	1.01 (0.68, 1.51)	0.96 (0.69, 1.35)	0.974	1.14 (0.75, 1.75)	1.12 (0.79, 1.61)	0.58	1.03 (0.69, 1.54)	1.01 (0.72, 1.41)	0.984
Thrombocytopenia	1.52 (1.10, 2.69)	1.00 (0.47, 1.35)	0.047	1.61 (1.16, 2.24)	1.10 (0.82, 1.48)	0.015	1.49 (1.08, 2.04)	0.96 (0.71, 1.29)	0.066
Macrocytosis	1.78 (1.18, 2.69)	1.60 (1.25, 2.06)	<0.001	1.66 (1.09, 2.53)	1.42 (1.10, 1.84)	0.003	1.76 (1.17, 2.66)	1.68 (1.30, 2.15)	<0.001
Cytopenia	1.82 (1.10, 3.01)	1.45 (0.97, 2.14)	0.022	1.96 (1.17, 3.30)	1.54 (1.03, 2.30)	0.009	1.81 (1.10, 2.99)	1.49 (1.01, 2.21)	0.019

*Note*: Model one estimates are adjusted for age, race, sex, and geographic region. Model two estimates are additionally adjusted for cancer history, smoking status, BMI, diabetes, alcohol consumption, CRP, and eGFR. Model three estimates are additionally adjusted for income, insurance status, and education level.

Abbreviations: CI, confidence interval; HR, hazard ratio.

^a^ p^int^ is the p‐value for the interaction between cytopenia and race in models having cancer death as outcome.

**TABLE 5:** Hazard ratios of individual hematologic parameters for death from hematologic neoplasia and death from solid tumor stratified by race

Hematologic parameter	Demographic model			Anemia/Cancer model			Socioeconomic model		
	(HR, 95%CI)			(HR, 95%CI)			(HR, 95%CI)		
	Blacks	Whites	*p* ^int^ ^a^	Blacks	Whites	*p* ^int^ ^a^	Blacks	Whites	*p* ^int^ ^a^
Hematologic neoplasia									
Anemia	2.84 (1.21, 6.66)	4.59 (2.68, 7.87)	<0.001	2.85 (1.17, 6.93)	4.54 (2.57, 8.02)	<0.001	2.77 (1.18, 6.51)	4.64 (2.70, 7.95)	<0.001
Leukopenia	1.37 (0.49, 3.79)	3.92 (2.29, 6.71)	<0.001	1.70 (0.60, 4.81)	3.94 (2.20, 7.05)	<0.001	1.38 (0.50, 3.82)	4.01 (2.34, 6.87)	<0.001
Thrombocytopenia	2.97 (1.43, 6.18)	2.34 (1.31, 4.17)	0.001	3.27 (1.55, 6.92)	2.50 (1.40, 4.47)	<0.001	2.91 (1.40, 6.07)	2.26 (1.26, 4.04)	0.002
Macrocytosis	3.24 (1.29, 8.16)	1.85 (0.98, 3.50)	0.019	3.54 (1.38, 9.13)	1.76 (0.90, 3.45)	0.023	3.27 (1.29, 8.25)	1.93 (1.02, 3.65)	0.015
Cytopenia	5.42 (2.93, 10.03)	5.06 (1.98, 12.89)	<0.001	5.70 (2.19, 14.80)	5.18 (2.72, 9.88)	<0.001	5.45 (2.94, 10.11)	5.07 (2.00, 12.88)	<0.001
Solid tumor									
Anemia	2.03 (1.42, 2.90)	1.44 (1.04, 2.00)	<0.001	2.14 (1.48, 3.10)	1.42 (1.01, 2.00)	<0.001	1.98 (1.39, 2.84)	1.39 (1.00, 1.93)	<0.001
Leukopenia	0.96 (0.62, 1.49)	0.59 (0.37, 0.93)	0.046	1.07 (0.67, 1.70)	0.72 (0.45, 1.16)	0.218	0.98 (0.63, 1.52)	0.62 (0.39, 0.97)	0.074
Thrombocytopenia	1.34 (0.94, 1.92)	0.81 (0.57, 1.15)	0.118	1.41 (0.98, 2.03)	0.89 (0.62, 1.27)	0.116	1.31 (0.92, 1.88)	0.77 (0.54, 1.10)	0.107
Macrocytosis	1.60 (1.01, 2.53)	1.56 (1.19, 2.05)	0.002	1.46 (0.91, 2.32)	1.37 (1.04, 1.81)	0.03	1.63 (1.24, 2.15)	1.57 (0.99, 2.49)	0.001
Cytopenia	1.41 (0.77, 2.58)	0.89 (0.52, 1.51)	0.508	1.47 (0.78, 2.77)	0.99 (0.58, 1.68)	0.445	1.40 (0.77, 2.56)	0.92 (0.54, 1.57)	0.545

*Note*: Model one estimates are adjusted for age, race, sex, and geographic region. Model two estimates are additionally adjusted for cancer history, smoking status, BMI, diabetes, alcohol consumption, CRP, and eGFR. Model three estimates are additionally adjusted for income, insurance status, and education level.

Abbreviations: CI, confidence interval; HR, hazard ratio.

^a^ *p* ^int^ is the *p*‐value for the interaction between cytopenia and race in models having cancer death as outcome.

## 4 DISCUSSION

In a large biracial and geographically diverse prospective cohort, a cytopenia phenotype was associated with 57%–67% increased risk of cancer mortality and constituted a race‐specific risk factor for cancer death, with an estimated 32%–42% higher risk in Black Americans compared to White Americans. The cytopenia‐associated cancer death and racial differences were not explained by demographic risk factors, socioeconomic risk factors, or risk factors for anemia and cancer.

There has been increasing understanding of the link between peripheral blood cell count abnormalities and cancer in recent years,^5^ through the identification of clonal hematopoiesis as a pre‐malignant state for hematologic cancers,^6^, ^8^ as well as carcinogenic pathways involving pro‐inflammatory responses in other organ systems, which create conditions that facilitate neoplastic transformation.^11^, ^12^ Furthermore, cytopenia directly attributable to malignancy can be present for years before other clinical manifestations establish the cancer diagnosis, especially in indolent hematologic malignancies.^48^, ^49^

Most studies exploring long‐term cancer‐related outcomes in individuals with cellular blood abnormalities have focused on anemia and its relationship to malignancy.^17^, ^18^ However, a cytopenia phenotype involving multiple cell lines and/or macrocytosis constitutes a better predictor of hematopoietic stress,^2^ while remaining widely available at low cost in routine care. Consequently, cytopenia should be considered a different clinical entity, with higher likelihood of coexisting with bone marrow suppression syndromes or chronic pro‐inflammatory states, and resultant long‐term health outcomes, including neoplasia. Due to prohibitive time and cost constraints of a prospective study, analysis of available longitudinal samples with a meticulous statistical methodology is the first necessary step to assess to value of cytopenia as a predictor of cancer mortality.

There is no standard definition for cytopenia in epidemiologic studies. Pancytopenia is associated with several hematologic and systemic conditions but may represent a later stage of more advanced disease. We previously demonstrated that cytopenia phenotype is associated with increased cardiovascular mortality,^33^ another outcome derived from the link between inflammation, carcinogenesis, and aging.^9^ In the current study, we demonstrate that cytopenia is associated with increased risk of cancer‐specific death. The association was found across models adjusting for age, race, sex, geographic region, cancer history, smoking, obesity, diabetes, alcohol use, inflammation markers, CKD, income, insurance status, and education. Though different phenotypes may have different associations with health outcomes, these findings suggest that cytopenia phenotype can predict an increased risk of cancer death which is not fully explained by coexisting traditional risk factors for cancer and/or overall poorer health outcomes. Our data agree with previous research showing an association of anemia with cancer death,^15^, ^19^ but further describes similar association patterns for other single cell line abnormalities (Table 2), proving the value of a combination cytopenia phenotype to predict cancer mortality.

The pathophysiologic mechanism behind the cytopenia and cancer death association remains to be fully defined and research is needed to identify potentially modifiable contributing factors. It is possible that baseline cytopenia limited the options for cancer treatment in affected subjects. For example, they could have received less intense chemotherapy to prevent further worsening of blood counts. Nevertheless, based on a stronger association with hematologic neoplasia than solid tumors (Table 3), we hypothesize that these findings are at least partially explained by undetected carcinogenic mutations in hematopoietic cells and clonal hematopoiesis. Hence, our data extend on previous epidemiologic reports calling for enhanced translation of genomic sequencing into clinical practice.^6^, ^30^ In individuals meeting the cytopenia phenotype, early detection could lead to future interventions to mitigate adverse long‐term outcomes, possibly including cancer mortality.

Racial disparities in cancer mortality are known, with increased cancer death rates in Black compared to White Americans across all cancer types.^50^ Cytopenia has not been previously studied as a marker of racial differences in cancer death. Nevertheless, Black Americans have a disproportionally high prevalence of anemia, which is not fully explained by disparities in socioeconomic status and comorbid conditions,^29^ and an NHANES study identified disproportionately high prevalence of unexplained cytopenia in non‐Hispanic Black American individuals.^30^ In the current study, the prevalence of cytopenia was not higher in Black participants. This is likely related to the use of race‐specific WBC thresholds to define cytopenia in Black participants (which has not been done in other population studies) and the high prevalence of macrocytosis seen in White older male participants. Still, our study is the first to analyze racial disparities in the cytopenia‐associated risk of cancer death at the population level.

We identified a significant race interaction in the association of cytopenia with cancer death (Table 4). This suggests that cytopenia exhibited a different effect on cancer mortality depending on race and likely indicates that cytopenia phenotype is a race‐specific risk factor for cancer mortality which affects Black more than White Americans. Furthermore, Black Americans not only had a higher risk of cancer death associated with cytopenia phenotype, but also similar findings for individual hematologic abnormalities including anemia and macrocytosis. Mediation analyses with at least two strategies to calculate indirect effect determined that cytopenia was not in the mediation pathway between race and cancer death. As cytopenia was not a mediator of the Black to White difference in cancer mortality, our results are evidence of unknown race‐specific factors linked to both cytopenia and cancer death.

Interestingly, the racial difference was not explained by predetermined risk factors included in our models. Thus, although we did not find a specific cause for the observed racial difference, our data prepare the way for further research aimed at decoding the pathophysiologic mechanisms linking race with cytopenia and cancer mortality. This is important because some factors behind the association could be modifiable, for example, quality of medical care and health behaviors are hypothesized as factors influencing cytopenia or cancer outcomes even after adjusting for comorbidities and socioeconomic factors.^29^, ^50^ Furthermore, these results could represent an epidemiologic pattern derived from racial variations which may exist within theorized mechanisms linking cytopenia and cancer death such as clonal hematopoiesis. For example, in our data, the largest racial difference was seen for risk of death from hematologic malignancy (Table 5), although lower numbers in subgroup analysis prevent general conclusions.

We acknowledge several limitations in our study. First, the observational study design cannot establish causality. Second, cytopenia was defined based on a single phlebotomy. Variations in hematologic parameters over time were not assessed but are known to be associated with adverse outcomes.^51^ Transient blood cell count abnormalities at study entry could have resulted in misclassification and overestimation of adverse outcomes associated with cytopenia. However, CBC was obtained during in‐home visits by trained professionals, and it is improbable that results were affected by unidentified transient illness, especially given the size of the study. Third, the cytopenia (exposure) prevalence was low and therefore our risk estimates were limited by low event rates. However, cytopenia prevalence and cancer death rates in our cohort are consistent with published population‐based studies.^30^ Fourth, a detailed list of prior cancer (for subjects with history of malignancy) or incident cancer during follow‐up is not available in REGARDS, including stage at initial cancer diagnosis during follow‐up. Lack of adjustment for cancer stage constitutes a limitation as we cannot rule out that cytopenia is a results of health care disparities leading to delays in diagnosis or more advanced stages at presentation. Fifth, not all secondary causes or risk factors for cytopenia and/or cancer were assessed. Unreported inflammatory comorbidities, behavioral factors, nutritional deficiencies, or conditions as autoimmune disease, immunosuppressive states, liver disease, and others could have interfered with results interpretation. To account for this limitation, we had a systematic approach across various models. Sensitivity analysis showed that results for cytopenia and race interactions were consistent when controlling for anemia/cancer risk factors. Furthermore, previous REGARDS studies have reported that nutritional deficiencies are rare,^52^ and that cytopenia is an independent phenotype from anemia, with no changes in cytopenia associations after adjusting for hemoglobin.^33^ Finally, the effect of unaccounted inflammation was minimized by adjusting for baseline CRP level. Nevertheless, despite effort to minimize confounding through sensitivity analyses and several multivariable models, we cannot rule out that the association between cytopenia and cancer death is not caused by unaccounted comorbidities or medications which cause cytopenia and constitute cancer risk factors.

Future studies in REGARDS will benefit from enhanced designs addressing some of these limitations. A successful linkage of primary REGARDS data with administrative Medicare claims has been previously conducted, which will allow analysis adjusting for specific comorbidities influencing the risk of cancer mortality. In addition, current efforts exist to link the data with State cancer registries, to account for incident cancer diagnosis and staging. Lastly, genomic analysis of biorepository samples from participants will allow to differentiate clonal hematopoiesis from other cytopenia etiologies.

Despite the limitations, our study strengths include a large sample size intentionally designed to identified racial differences in health outcomes, uniform laboratory analysis of CBC samples, active prospective cohort monitoring, a long follow‐up period, and a rigorous adjudication process for identification of cancer mortality including hospital records review and national registries. These characteristics permitted the largest study to date evaluating the association of cytopenia with cancer death and its related racial differences. Our results can inform future research to identify mechanisms that explain the racial interaction in the cytopenia to cancer death association, which may be clinically relevant and a potential target to improve cancer outcomes.

## 5 CONCLUSION

In a large biracial cohort, cytopenia phenotype was associated with increased risk of cancer mortality and constituted a race‐specific risk factor for cancer death, with stronger association in Black compared to White participants. Race was an effect modifier in the association of cytopenia and cancer mortality, with different cytopenia effect on cancer mortality risk depending on race. However, cytopenia was not a mediator of the Black to White difference in cancer death. The limitations of our cohort cannot establish causality and the association between cytopenia and cancer death may be indirect. Thus, further research is needed to confirm the racial interaction in the cytopenia to cancer death link and identify the factors contributing to racial disparities in cancer mortality.

## AUTHOR CONTRIBUTIONS

**Diego Andres Adrianzen‐Herrera:** Conceptualization (lead); data curation (lead); formal analysis (lead); funding acquisition (lead); investigation (lead); methodology (lead); project administration (equal); resources (lead); software (lead); supervision (lead); validation (lead); visualization (lead); writing – original draft (lead); writing – review and editing (lead). **Insu Koh:** Data curation (equal); formal analysis (equal); methodology (equal); software (equal). **Radhika Gangaraju:** Conceptualization (equal); data curation (supporting); methodology (equal); validation (equal); writing – review and editing (supporting). **Tomi F Akinyemiju:** Data curation (equal); funding acquisition (equal); methodology (supporting); resources (supporting); supervision (supporting). **Neil Zakai:** Conceptualization (equal); formal analysis (supporting); methodology (equal); project administration (supporting); supervision (supporting); writing – review and editing (supporting).

## FUNDING INFORMATION

The REasons for Geographic And Racial Differences in Stroke (REGARDS) study was supported by cooperative agreement U01 NS041588 co‐funded by the National Institute of Neurological Disorders and Stroke (NINDS) and the National Institute on Aging (NIA), National Institutes of Health, Department of Health and Human Service. Cancer mortality outcomes were obtained from grant R01 HL80477 and K08 HL096841 from the National Heart, Lung, Blood Institute. The content is solely the responsibility of the authors and does not necessarily represent the official views of the NINDS or the NIA. Representatives of the funding agencies were involved in the review of the manuscript but were not directly involved in the collection, management, analysis, or interpretation of the data.

## CONFLICT OF INTEREST

RG serves as a Consultant for Sanofi Genzyme and Alexion. The authors have no other conflicts of interest to declare.

## Supporting information


Figure S1.

Table S1.

Table S2.

Table S3.

Table S4.

## References

[1] Seidel MG . Autoimmune and other cytopenias in primary immunodeficiencies: pathomechanisms, novel differential diagnoses, and treatment. Blood. 2014;124(15):2337‐2344. 25163701 10.1182/blood-2014-06-583260 PMC4192747

[2] Gnanaraj J , Parnes A , Francis CW , Go RS , Takemoto CM , Hashmi SK . Approach to pancytopenia: diagnostic algorithm for clinical hematologists. Blood Rev. 2018;32(5):361‐367. 29555368 10.1016/j.blre.2018.03.001

[3] Valent P . Low blood counts: immune mediated, idiopathic, or myelodysplasia. Hematology Am Soc Hematol Educ Program. 2012;2012:485‐491. 23233623 10.1182/asheducation-2012.1.485

[4] Valent P , Bain BJ , Bennett JM , et al. Idiopathic cytopenia of undetermined significance (ICUS) and idiopathic dysplasia of uncertain significance (IDUS), and their distinction from low risk MDS. Leuk Res. 2012;36(1):1‐5. 21920601 10.1016/j.leukres.2011.08.016

[5] DeZern AE , Sekeres MA . The challenging world of cytopenias: distinguishing myelodysplastic syndromes from other disorders of marrow failure. Oncologist. 2014;19(7):735‐745. 24899643 10.1634/theoncologist.2014-0056 PMC4077450

[6] Malcovati L , Galli A , Travaglino E , et al. Clinical significance of somatic mutation in unexplained blood cytopenia. Blood. 2017;129(25):3371‐3378. 28424163 10.1182/blood-2017-01-763425 PMC5542849

[7] DeZern AE , Malcovati L , Ebert BL . CHIP, CCUS, and other acronyms: definition, implications, and impact on practice. Am Soc Clin Oncol Educ Book. 2019;39:400‐410. 31099654 10.1200/EDBK_239083

[8] Steensma DP , Bejar R , Jaiswal S , et al. Clonal hematopoiesis of indeterminate potential and its distinction from myelodysplastic syndromes. Blood. 2015;126(1):9‐16. 25931582 10.1182/blood-2015-03-631747 PMC4624443

[9] Balducci L . Anemia, cancer, and aging. Cancer Control. 2003;10(6):478‐486. 14652524 10.1177/107327480301000606

[10] Mao M , Wei X , Sheng H , et al. C‐reactive protein/albumin and neutrophil/lymphocyte ratios and their combination predict overall survival in patients with gastric cancer. Oncol Lett. 2017;14(6):7417‐7424. 29344182 10.3892/ol.2017.7179 PMC5755031

[11] Hussain SP , Harris CC . Inflammation and cancer: an ancient link with novel potentials. Int J Cancer. 2007;121(11):2373‐2380. 17893866 10.1002/ijc.23173

[12] Federico A , Morgillo F , Tuccillo C , Ciardiello F , Loguercio C . Chronic inflammation and oxidative stress in human carcinogenesis. Int J Cancer. 2007;121(11):2381‐2386. 17893868 10.1002/ijc.23192

[13] Hasselbalch HC . Perspectives on chronic inflammation in essential thrombocythemia, polycythemia vera, and myelofibrosis: is chronic inflammation a trigger and driver of clonal evolution and development of accelerated atherosclerosis and second cancer? Blood. 2012;119(14):3219‐3225. 22318201 10.1182/blood-2011-11-394775

[14] Gaspar BL , Sharma P , Das R . Anemia in malignancies: pathogenetic and diagnostic considerations. Hematology. 2015;20(1):18‐25. 24666207 10.1179/1607845414Y.0000000161

[15] Spivak JL . The anaemia of cancer: death by a thousand cuts. Nat Rev Cancer. 2005;5(7):543‐555. 15965494 10.1038/nrc1648

[16] Guralnik JM , Eisenstaedt RS , Ferrucci L , Klein HG , Woodman RC . Prevalence of anemia in persons 65 years and older in the United States: evidence for a high rate of unexplained anemia. Blood. 2004;104(8):2263‐2268. 15238427 10.1182/blood-2004-05-1812

[17] Means RT Jr . Recent developments in the anemia of chronic disease. Curr Hematol Rep. 2003;2(2):116‐121. 12901142

[18] Atkin C , Sapey E , Richter A . Change in blood test results prior to diagnosis in multiple myeloma. Clin Med (Lond). 2020;20(Suppl 2):s99‐s100. 32409406 10.7861/clinmed.20-2-s99 PMC7243514

[19] Edgren G , Bagnardi V , Bellocco R , et al. Pattern of declining hemoglobin concentration before cancer diagnosis. Int J Cancer. 2010;127(6):1429‐1436. 20020493 10.1002/ijc.25122

[20] Riedl J , Posch F , Konigsbrugge O , et al. Red cell distribution width and other red blood cell parameters in patients with cancer: association with risk of venous thromboembolism and mortality. PLoS One. 2014;9(10):e111440. 25347577 10.1371/journal.pone.0111440 PMC4210186

[21] Li J , Yang X , Ma J , Gong F , Chen Q . Relationship of red blood cell distribution width with cancer mortality in hospital. Biomed Res Int. 2018;2018:8914617. 30539025 10.1155/2018/8914617 PMC6261390

[22] Ghanavat M , Ebrahimi M , Rafieemehr H , Maniati M , Behzad MM , Shahrabi S . Thrombocytopenia in solid tumors: prognostic significance. Oncol Rev. 2019;13(1):413. 31205603 10.4081/oncol.2019.413 PMC6542370

[23] Lim E , Miyamura J , Chen JJ . Racial/ethnic‐specific reference intervals for common laboratory tests: a comparison among Asians, Blacks, Hispanics, and White. Hawaii J Med Public Health. 2015;74(9):302‐310. 26468426 PMC4578165

[24] Hsieh MM , Everhart JE , Byrd‐Holt DD , Tisdale JF , Rodgers GP . Prevalence of neutropenia in the U.S. population: age, sex, smoking status, and ethnic differences. Ann Intern Med. 2007;146(7):486‐492. 17404350 10.7326/0003-4819-146-7-200704030-00004

[25] Coates S , Wang D , Pierscionek T , et al. Time‐ and race‐specific Haematological reference intervals for healthy volunteer trials: a retrospective analysis of pooled data from multiple phase I trials. Front Pharmacol. 2020;11:314. 32231575 10.3389/fphar.2020.00314 PMC7082321

[26] Beutler E , West C . Hematologic differences between African‐Americans and whites: the roles of iron deficiency and alpha‐thalassemia on hemoglobin levels and mean corpuscular volume. Blood. 2005;106(2):740‐745. 15790781 10.1182/blood-2005-02-0713 PMC1895180

[27] Lakhotia R , Aggarwal A , Link ME , Rodgers GP , Hsieh MM . Natural history of benign ethnic neutropenia in individuals of African ancestry. Blood Cells Mol Dis. 2019;77:12‐16. 30909074 10.1016/j.bcmd.2019.01.009 PMC6541485

[28] Yang F , Liu X , Zha P . Trends in socioeconomic inequalities and prevalence of anemia among children and nonpregnant women in low‐ and middle‐income countries. JAMA Netw Open. 2018;1(5):e182899. 30646183 10.1001/jamanetworkopen.2018.2899 PMC6324516

[29] Zakai NA , McClure LA , Prineas R , et al. Correlates of anemia in American blacks and whites: the REGARDS renal ancillary study. Am J Epidemiol. 2009;169(3):355‐364. 19066309 10.1093/aje/kwn355 PMC2720717

[30] Alpert N , Rapp JL , Mascarenhas J , et al. Prevalence of Cytopenia in the general population‐a National Health and nutrition examination survey analysis. Front Oncol. 2020;10:579075. 33330056 10.3389/fonc.2020.579075 PMC7714991

[31] Zakai NA , Katz R , Hirsch C , et al. A prospective study of anemia status, hemoglobin concentration, and mortality in an elderly cohort: the cardiovascular health study. Arch Intern Med. 2005;165(19):2214‐2220. 16246985 10.1001/archinte.165.19.2214

[32] Denny SD , Kuchibhatla MN , Cohen HJ . Impact of anemia on mortality, cognition, and function in community‐dwelling elderly. Am J Med. 2006;119(4):327‐334. 16564775 10.1016/j.amjmed.2005.08.027

[33] Gangaraju R , Koh I , Irvin MR , et al. Peripheral blood cytopenia and risk of cardiovascular disease and mortality. J Am Heart Assoc. 2021;10:e020809. 34514816 10.1161/JAHA.121.020809 PMC8649504

[34] Howard VJ , Cushman M , Pulley L , et al. The reasons for geographic and racial differences in stroke study: objectives and design. Neuroepidemiology. 2005;25(3):135‐143. 15990444 10.1159/000086678

[35] Gillett SR , Boyle RH , Zakai NA , McClure LA , Jenny NS , Cushman M . Validating laboratory results in a national observational cohort study without field centers: the reasons for geographic and racial differences in stroke cohort. Clin Biochem. 2014;47(16‐17):243‐246. 25130959 10.1016/j.clinbiochem.2014.08.003 PMC5038129

[36] Reed WW , Diehl LF . Leukopenia, neutropenia, and reduced hemoglobin levels in healthy American blacks. Arch Intern Med. 1991;151(3):501‐505. 2001132

[37] Saxena S , Wong ET . Heterogeneity of common hematologic parameters among racial, ethnic, and gender subgroups. Arch Pathol Lab Med. 1990;114(7):715‐719. 2363629

[38] Beutler E , Waalen J . The definition of anemia: what is the lower limit of normal of the blood hemoglobin concentration? Blood. 2006;107(5):1747‐1750. 16189263 10.1182/blood-2005-07-3046 PMC1895695

[39] Charles BA , Hsieh MM , Adeyemo AA , et al. Analyses of genome wide association data, cytokines, and gene expression in African‐Americans with benign ethnic neutropenia. PLoS One. 2018;13(3):e0194400. 29596498 10.1371/journal.pone.0194400 PMC5875757

[40] Babitt JL , Lin HY . Mechanisms of anemia in CKD. J Am Soc Nephrol. 2012;23(10):1631‐1634. 22935483 10.1681/ASN.2011111078 PMC3458456

[41] Akinyemiju T , Moore JX , Pisu M , et al. A prospective study of dietary patterns and cancer mortality among blacks and whites in the REGARDS cohort. Int J Cancer. 2016;139(10):2221‐2231. 27459634 10.1002/ijc.30287 PMC5041524

[42] Safford MM , Brown TM , Muntner PM , et al. Association of race and sex with risk of incident acute coronary heart disease events. JAMA. 2012;308(17):1768‐1774. 23117777 10.1001/jama.2012.14306 PMC3772637

[43] Huntington JT , Butterfield M , Fisher J , Torrent D , Bloomston M . The social security death index (SSDI) most accurately reflects true survival for older oncology patients. Am J Cancer Res. 2013;3(5):518‐522. 24224129 PMC3816971

[44] Quinn J , Kramer N , McDermott D . Validation of the social security death index (SSDI): an important readily‐available outcomes database for researchers. West J Emerg Med. 2008;9(1):6‐8. 19561695 PMC2672222

[45] Curb JD , Ford CE , Pressel S , Palmer M , Babcock C , Hawkins CM . Ascertainment of vital status through the National Death Index and the Social Security Administration. Am J Epidemiol. 1985;121(5):754‐766. 4014167 10.1093/aje/121.5.754

[46] Tchetgen Tchetgen EJ . Inverse odds ratio‐weighted estimation for causal mediation analysis. Stat Med. 2013;32(26):4567‐4580. 23744517 10.1002/sim.5864 PMC3954805

[47] Yu Q , Wu X , Li B , Scribner RA . Multiple mediation analysis with survival outcomes: with an application to explore racial disparity in breast cancer survival. Stat Med. 2019;38(3):398‐412. 30255567 10.1002/sim.7977 PMC6320301

[48] Landgren O , Albitar M , Ma W , et al. B‐cell clones as early markers for chronic lymphocytic leukemia. N Engl J Med. 2009;360(7):659‐667. 19213679 10.1056/NEJMoa0806122 PMC7015348

[49] Landgren O , Kyle RA , Pfeiffer RM , et al. Monoclonal gammopathy of undetermined significance (MGUS) consistently precedes multiple myeloma: a prospective study. Blood. 2009;113(22):5412‐5417. 19179464 10.1182/blood-2008-12-194241 PMC2689042

[50] The Lancet Oncology . Racial disparities in cancer care: can we close the gap? Lancet Oncol. 2021;22(12):1643. 34856134 10.1016/S1470-2045(21)00669-0

[51] Zakai NA , French B , Arnold AM , et al. Hemoglobin decline, function, and mortality in the elderly: the cardiovascular health study. Am J Hematol. 2013;88(1):5‐9. 23044913 10.1002/ajh.23336 PMC3860593

[52] Kancherla V , Garn JV , Zakai NA , et al. Multivitamin use and serum vitamin B12 concentrations in older‐adult metformin users in REGARDS, 2003‐2007. PLoS One. 2016;11(8):e0160802. 27513580 10.1371/journal.pone.0160802 PMC4981300

