# Supplementary figures and images for: Association between peripheral blood cytopenia and cancer mortality: A race‐specific risk factor for cancer death
Source: Cancer Med. 2022 Dec 30;12(7):8639–51. doi: 10.1002/cam4.5570 (PMC10134255; doi:10.1002/cam4.5570)

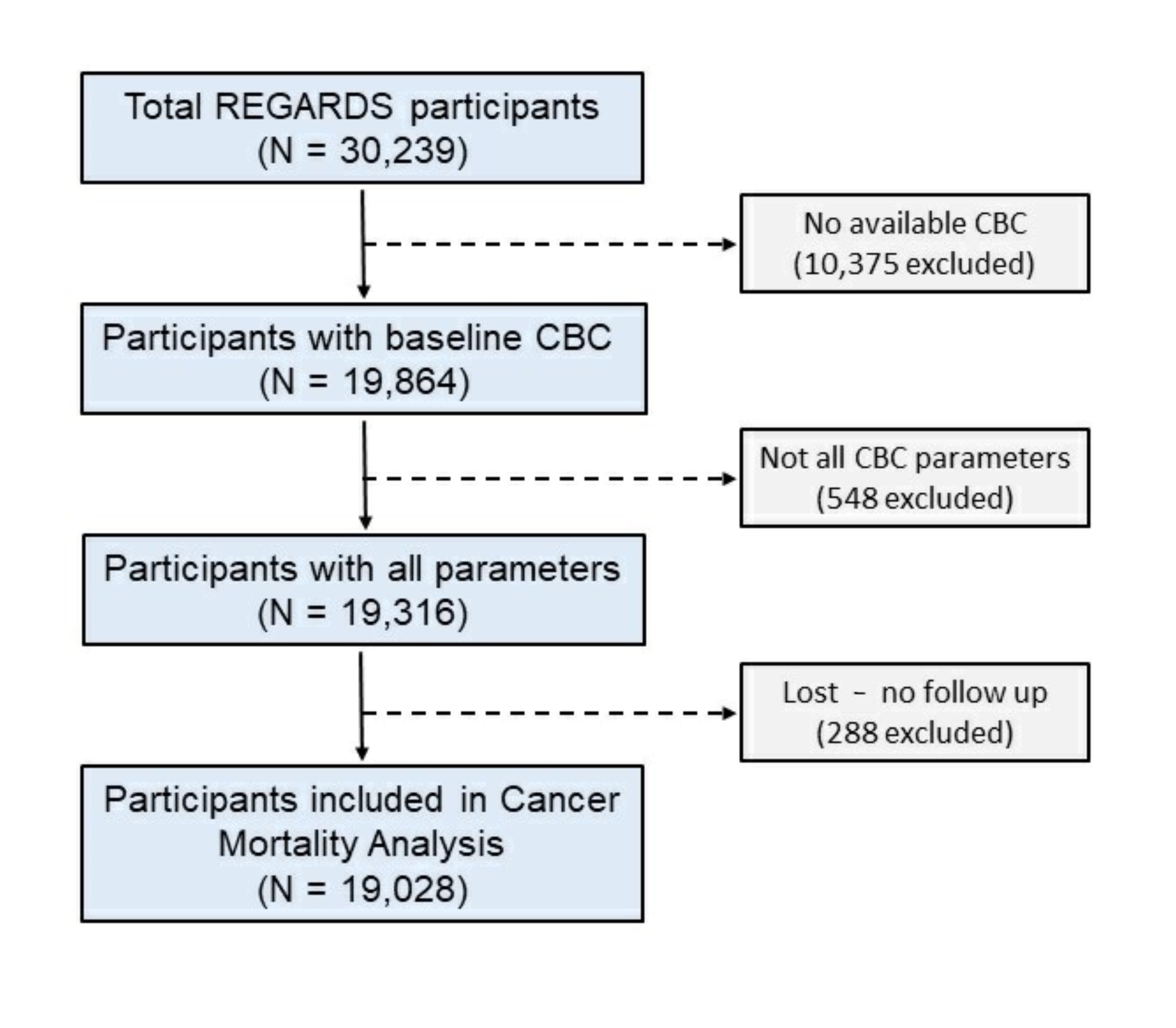

Supplement: Supplementary file 1 — Figure S1. [file CAM4-12-8639-s005.tif]
